# Agree to Disagree? Fertility Intentions Among Mixed Couples in Sweden

**DOI:** 10.1007/s10680-025-09742-w

**Published:** 2025-08-06

**Authors:** Eleonora Mussino, Caroline Uggla

**Affiliations:** 1 Stockholm University Demography Unit (SUDA), Stockholm, Sweden; 2 Umeå University, Umeå, Sweden; 3 Södertörn University, Huddinge, Sweden

**Keywords:** Fertility intentions, Couple perspective, Migrants, Mixed unions, GGS, Sweden

## Abstract

Whether couples agree on having a(nother) child is crucial for both individuals and society. While fertility research has long focused on women, recent studies emphasize the need to incorporate both partners’ perspectives. However, analyses that jointly consider men’s and women’s fertility intentions remain scarce. This focus on women has been partly driven by homogamy—the tendency for individuals to select partners with similar traits and values. Given that couples with mixed backgrounds have higher dissolution rates, they may also be less likely to share family-related beliefs. This study examines how agreement on fertility intentions varies among mixed and homogamous couples in Sweden. Using the 2021 Swedish Generation and Gender Survey (GGS) and stratifying by respondents’ gender, we find that most couples agree not to have a(nother) child, reflecting recent fertility declines. Couples where both partners are migrants exhibit the highest agreement, while mixed couples show the most disagreement and the strongest gender asymmetries in reported intentions. However, these differences are small and vary by the gender of the reporting partner. The higher disagreement among mixed couples aligns with broader research on their elevated dissolution risks. However, reverse causality is possible—value differences may be linked to other stressors, making childbearing less desirable. By highlighting the role of couple composition in fertility decision-making, our findings contribute to understanding how family formation dynamics vary across different couple types.

## Introduction

That men matter for reproductive decision-making is well established in international scholarship (Ryder, 1973). Numerous researchers have embraced a couple-centred framework in fertility studies (Beckman et al., 1983; Coombs & Chang, 1981; Fraboni & Rosina, 2006; Fried & Udry, 1979; Jansen & Liefbroer, 2006; Morgan, 1985; Rijken & Thomson, 2011; Testa, 2012; Thomson, 1997; Thomson & Hoem, 1998). Despite this, most fertility research still centres on the female perspective. A female focus is often rationalized by the significant level of similarity within couples, and by the role of women as both the principal participants in and the most accurate reporters of procreative events in heterosexual couples. However, if these assumptions no longer hold, overlooking male views may have serious consequences.

Research on partner choice in Europe in recent decades indeed provides reasons to question the validity of these assumptions. First, there is much evidence to suggest that the degree of similarity within couples has changed in recent years. Homogamy is the tendency for individuals to pair with partners who share similar socioeconomic, demographic, and cultural traits, such as age, education, race, and religion (Blackwell & Lichter, 2004; Kalmijn, 1991; van Poppel et al., 2001). Homogamy has increased over time in terms of both educational attainment and age (Schwartz & Mare, 2005; Van De Putte et al., 2009). However, alongside the growing homogamy on some traits, there has been a notable divergence in the country of origin of partners. This shift has been driven by the rising prevalence of intermarriages between natives and immigrants. When more couples come from different country backgrounds, the assumption of within-couple similarity might not hold as often. Focusing solely on one person’s childbearing intentions (often the woman’s) becomes a more problematic issue when the two people in the couple are less likely to share family beliefs. Age at first childbearing and number of children might vary considerably from the norm of natives in the destination country. When native-immigrant couples are formed, will these individuals face more disagreement in family decision-making? Or are couples self-selecting on family values so that there is no difference, or even lower disagreement compared to couples consisting only of natives or only of immigrants? The diversification of couple composition, especially in the context of increasing international mobility and multicultural societies, warrants a broader approach that encompasses both partners’ perspectives to fully understand reproductive decision-making.

There is also reason to question the assumption of women having the main say over reproductive decision-making. Women do have greater influence over reproductive decision-making *when* childbearing is in the women’s interest (Fried et al., 1980; Miller & Pasta, 1996). After the second demographic transition, Beckerian specialization of labour market vs. childbearing spheres (Becker, 1981) is less pertinent in many contexts. Men and women may instead both contribute significantly to household earnings and to childrearing tasks in a dual earner model. With more gender equality, men may be more involved and their views matter more for the decision to have a(nother) child.

This paper compares the (perceived) agreement on fertility intentions among mixed couples and couples consisting of two natives or two immigrants. While those in exogamous unions constitute a relatively small subset of overall populations, their experiences offer profound insights into fertility decision-making. Exploring the dynamics within intermarried couples sheds light on reproductive choices in contexts where traditional homogamy does not hold. In this respect, intermarried couples present an intriguing case study. Examination of fertility intentions among mixed couples is critical, as it challenges the assumptions of the predominant research focus on women by bringing forth the complex interplay of factors influencing the fertility decisions in couples with diverse backgrounds. This approach can reveal the nuances and broader implications of reproductive decision-making in increasingly heterogeneous societies.

Additionally, in this paper, we use survey data from Sweden to explore whether agreement on *a)* having a(nother) birth, *b)* not having a(nother) birth or c) disagreement on having a birth varies by couple’s migrant background. The ability to distinguish between couple’s attitude towards having or not having a(nother) child brings a broader contribution to understand fertility behaviours beyond merely assessing determinants of attitude homogeneity among couples.

## Theoretical Background

### Fertility Intentions and (Dis)agreement

People’s intentions to have a child is a topic of large demographic relevance in a wide range of contexts. As fertility is declining to record low levels in many parts of the world, demographers are seeking to understand the many factors that contribute to delaying or foregoing children completely. Partnership instability may be a contributing factor to lower fertility, but even within stable partnerships disagreement may lead to delayed or lower levels of childbearing overall. Couples may agree to have a child/continue having children, agree to not have any more children or disagree (one person holding a positive intention and the other not). Disagreement on whether to have a birth in the future is common. Doepke and Kindermann (2019) using Gender and Generation Survey (GGS) data across 19 countries report that 25–50% of all couples where at least one partner wants a baby, the other partner does not. Among couples who have two children, more couples disagree about a third birth than those where both agree. The causes of disagreement are important to understand as they are related to relationship satisfaction, wellbeing, relationship dissolution—and actual fertility. Mixed unions have never, to our knowledge, been studied in this perspective. Examining this topic has both academic and societal relevance given increasing prevalence of mixed unions and falling fertility rates.

In this study, we examine the determinants of (dis)agreement within couples. Yet, it is important to note that couples can resolve disagreement on whether to have a(nother) child in various ways. The *Golden mean rule* states that couples view each other’s views as equally important and try to find a mean number of children or intermediate time to next birth. Support for this perspective has been found both in the Netherlands (Jansen & Liefbroer, 2006), USA (Thomson, 1997) and Sweden (Thomson & Hoem, 1998). The social drift rule states that maintenance of the status quo favours the partner who does not want a(nother) child. Other ways to resolve dissimilar intentions may be through bargaining. A *bargaining or power perspective* asserts that the partner with the most access to resources will prevail in the reproductive decision-making (Jansen & Liefbroer, 2006). It is also possible that the person with the largest sphere of influence in the childbearing domain (often the woman) has more say in the decision.

Both what kind of fertility intentions individuals hold, and whether they are materialised, vary by the social context. In Sweden, a positive intention of both partners is important for entering parenthood, but *women’s* intentions are more influential when it comes to second and third births (Duvander et al., 2020). Doepke and Kindermann (2019) find that in low fertility countries in Europe, women are more likely to disagree with their partners’ intention for a child, whereas there is no gender difference in countries with higher fertility.

What is currently known in a European context about the determinants of disagreement on fertility intentions within couples comes mainly from Italy and Austria. In Austria, Testa found that men without children are more responsive to the perception of their partner’s fertility desires than childless women (Testa, 2012). In her study on disagreement among Italian couples with one child, Cavalli (2012) saw that disagreement increased with a woman’s age (especially if she is older than 40 years). However, among Italian couples, disagreement is not very different between childless couples and those who already had had one or two children (Testa et al., 2014). Gender equality may also matter. Italian women in more egalitarian unions are more likely to disagree with their male partner to have a first or second child at any given time (Cavalli, 2012; Cavalli & Rosina, 2011; Rosina & Testa, 2009). These women are less likely to agree with a male partner to have a first child if they are living in unmarried cohabitation, are highly educated and employed. Not being satisfied with the division of housework within the couple is also a strong predictor of disagreeing to have a first child (Rosina & Testa, 2009). Unequal division of household chores has been found to affect not only the fertility intention negatively, but also the realizations of that intention across four European countries (Riederer et al., 2019).

### Fertility and Fertility Intentions of Migrants

When examining disagreement on fertility intentions in mixed couples, it is useful to briefly review what we know about fertility intentions of migrants compared to the native population. In Italy, migrant women have higher positive intentions for a child within 3 years, and lower negative intention than native Italian women (Mussino et al., 2021a). Previous studies of fertility intentions among Swedish migrants have shown that migrants from the Middle East, Northern Africa, and Eastern Europe have elevated intentions compared to natives (Carlsson, 2018). Notably, these findings on intentions do not map completely with other research on actual fertility between migrant groups; some migrant groups with higher fertility intentions have lower actual fertility than native Swedes (Andersson et al., 2017; Scott & Stanfors, 2011). Generally, fertility and fertility intentions decrease with economic constraints and uncertainty (Fiori et al., 2014), and it is common that migrants face such issues, especially in the first years after arriving in the host country.

Over time, there is convergence in fertility intentions of migrants to the native norm. However, such convergence is not uniform but may vary by gender and origin group. In Sweden, immigrant women arriving as children (the so-called “1.5 generation”) have fertility intentions close to non-migrants, whereas for men, no intergenerational trend was found. Eastern Europeans show convergence, whereas the same is not true for Middle Eastern and Northern African migrants (Carlsson, 2018).

Overall, migrants have elevated birth risks after migration (Andersson, 2004), in part because international relocation puts a halt to family formation processes. In Sweden, timing of first birth shows adaptation over time, so that migrants who arrive at younger ages are more likely to behave like the native Swedish population (Mussino et al., 2021b). Overall, fertility levels do not show the same adaptation, however (Mussino et al., 2021b).

It is worth pointing out that another driver of fertility intentions is the sex of existing children. Not having had at least one child of either sex or the preferred sex may be a reason to extend the family, for natives and immigrants alike. Interestingly, the majority population in Sweden holds a daughter preference (Andersson et al., 2006; Miranda et al., 2018), whereas many immigrant groups have a clear son preference (Mussino et al., 2018, 2019).

### Mixed Unions

Intermarriage is often used as the ultimate sign of integration. When two people from different ethnic groups marry, it might signal acceptance at the group level (Kulu & González-Ferrer, 2014) and indicate that groups view each other as equals (Kalmijn, 1991). Yet, it is far from certain that individuals in a mixed couple share views in all domains. In various contexts, mixed (or ethnically exogamous) unions are more likely to dissolve than others (Dribe & Lundh, 2012). Higher dissolution rates in mixed couples could be caused by a range of factors, such as social sanctions from breaking a social norm, lesser support from kin if relatives are more distant, or from stressors such as discrimination or integration difficulties. However, given that individuals have been socialized in different contexts, it is also possible that holding different views about family life in general, or fertility decision-making in particular, might contribute to union dissolution.

Studies of native–migrant unions have uncovered some asymmetries in other partner traits. For instance, immigrants of either sex are more attractive on the partner market if they are younger than native spouses (Elwert, 2020). It is also more likely that natives who enter mixed unions are characterized by lower education and income and are older (Haandrikman, 2014; Niedomysl et al., 2010). Immigrants in Sweden tend to partner with others who are more similar to themselves in terms of values (Dribe & Lundh, 2008) and less likely to dissolve such unions (Dribe & Lundh, 2012). Overall, immigrants who have higher levels of education, who do not reside in large cities, and who have lived longer in Sweden before marriage are more likely to be married to a native Swedish person than other immigrants (Dribe & Lundh, 2008). Moreover, individuals who have been in an intermarriage are more likely to enter another intermarriage than those who previously had a endogamous marriage (Obućina, 2016). Swedish-born people who have at least one migrant parent are more likely to intermarry than Swedish-born with two Swedish-born parents (Irastorza & Elwert, 2019). Some of the underlying demographic and social characteristics of mixed couples can contribute to understanding dis(agreement) among these couples.

While the union dissolution of mixed couples has been extensively studied, we know almost nothing about their fertility and fertility intentions. An exception is Elwert (2023) who demonstrated that having a third birth is more likely in couples consisting of Swedish women and immigrant men. Unions between Swedish men and immigrant women, on the other hand, are less likely to have a third birth (Elwert, 2023). As far as we know, no study has examined differences in intentions between mixed couples and native couples or migrant–migrant couples.

## Why Might There be more (Dis)agreement Among Mixed Couples?

There are many reasons why partners may not agree on whether to have a(nother) child or disagree on the timing of that pregnancy. Here we discuss specifically why agreement on fertility intentions may differ between native–migrant couples and couples where both individuals are either native or a migrant. First, cultural norms regarding desired family size and the timing of childbearing often vary between natives and migrants. If their ideational views on these aspects differ, this may translate to different short-term fertility intentions within a mixed couple. Migrants and Swedes experience distinct *socialization* processes that shape their family ideals, influencing how they wish to organize family life. Since native–native and migrant–migrant couples have typically been raised in similar contexts with shared expectations, they are more likely to align on future family plans compared to intermarried couples, who may face greater ideological and practical differences. This is consistent with research on divorce, which finds that intermarried couples have higher dissolution risks.

Second, mixed couples tend to have higher rates of union dissolution than couples who share the same country of birth. If the union is perceived as more labile, it is possible that this puts doubts in at least one person’s mind about having a(nother) common child. Causality is tricky here; it could be that disagreeing on fertility intentions leads to dissolution rather than the other way around. However, if mixed couples hold more dissimilarity in other values than non-mixed couples, different values can be causing union instability and therefore also fertility disagreement.

Third, partners who were not raised with similar cultural norms and values may experience third-party influence disapproving of their union (Hohmann-Marriott & Amato, 2008; Killian, 2001). If this is the case, it is more likely that there is hesitation from at least one person to proceed with a(nother) birth, compared to couples who are not subject to any type of social sanctions.

Fourth, because mixed unions consist of at least one migrant person, mixed unions may have fewer kin nearby than native couples. With a less stable social support network, there could be more negative fertility intentions or a disagreement about fertility. If this is true, we might expect even high disagreement among migrant–migrant couples.

In contrast, it is also possible that there are no differences between mixed couples, native couples and couples consisting of two migrants. First, processes of adaptation mean that migrants adopt the family norms and practices of the destination country over time. Adaptation of age at first birth in Sweden has been observed for several groups (Mussino et al., 2021b) and so this may mean that mixed couples are not too dissimilar in their views on when to start having children or how many children to have. Second, if there is strong selection into partnerships, individuals within mixed unions can be expected to share fertility intentions, despite having different countries of birth. One could argue that mixed couples may actually exhibit higher agreement on fertility intentions if they are more likely to self-select partners who share their life goals. In line with this, intermarried immigrants are often described as positively selected based on observable characteristics (Dribe & Lundh, 2008) and are likely to be selected in terms of unobserved characteristics, including fertility preferences (González-Ferrer, 2006). Consequently, fertility patterns resembling those of the Swedish majority may not be a direct outcome of intermarriage but rather a reflection of selection into mixed unions. Similarly, intermarried native individuals may constitute a select group whose fertility preferences differ from those of the native majority. Therefore, we could expect intermarried couples to exhibit greater agreement on their fertility plans.

However, we take into account that couples may agree on a positive intention or on a negative intention. Fertility intentions can be categorized in three ways: the couple could a) agree to have a(nother) birth, b) agree to *not* have a(nother) birth, or they could disagree on whether to have more children.

## The Swedish Context

Sweden has a large and diverse population of immigrants due to a long history of sustained levels of immigration. Since the latter half of the twentieth century, the migrant population in Sweden has grown considerably. As the predominant form of migration has changed from labour migration during the 1950s and 60s to refugee migration during the late 1970s, 1980s, and 1990s, migration has also become more diverse. Currently, about a fourth of the Swedish population are either migrant or have at least one migrant parent (Statistics Sweden 2022). The number of mixed unions in Sweden has increased since the 1970s and the increase comes mainly from marriages to non-EU citizens (Haandrikman, 2014).

Sweden is characterised by high levels of gender equality and is, alongside other Nordic countries, often considered a forerunner in family change with high levels of female labour force participation, paternity leave, and gender equality (Bernhardt et al., 2007). In a gender equal society, male and female immigrants should have the same opportunities, at least with respect to Swedish institutions and society at large. For instance, Sweden has generous family policies and provides considerable support to parents, with paid parental leave and subsidised childcare which can affect couple dynamics. In addition, it has had comparatively high levels of fertility. For the time period of the GGS, the TFR was around 1.6 and it has now fallen further to a record low of just above 1.4 children per woman (Statistics Sweden 2024). For childbearing intentions, high gender equality means that both causes for disagreement and how it is handled within couples are likely to differ from contexts where childbearing still is placed firmly within a female sphere.

## This Study

In this study, we examine partners’ agreement or disagreement on short-term fertility intentions—whether to have a child or not—and distinguish responses by gender of the respondent. This distinction is important because gendered differences in fertility preferences, communication patterns, and decision-making power may influence how agreement or disagreement is reported. Our main exploratory dimension is the migrant background of the couple, distinguishing between both native, mixed, and both migrant couples. While we acknowledge that differentiating by specific country backgrounds could provide further insight, our survey data do not allow for such fine-grained analyses.

To the best of our knowledge, no previous studies have examined differences between mixed and endogamous couples in their agreement or disagreement on having a child. Thus, our study serves as a valuable first step in addressing this gap, using a descriptive approach to categorize couples as mixed or non-mixed (native–native or migrant–migrant). Here we use GGS survey data on fertility intentions among couples in Sweden to answer the question: Do mixed couples disagree more or less than native and migrant couples?

## Data and Method

We utilize the 2021 iteration of the Swedish Generation and Gender Survey (GGS), a national survey designed to reflect the Swedish population. This survey, conducted under the broader Generation and Gender Program (GGP), investigates various stages and dynamics of the family lifecycle, including relationships, marriages, parenthood, and divorces, as well as the accompanying societal and personal challenges. Notably, the 2021 GGS marks the second round of data collection in Sweden, following the inaugural round in 2012.

Our research specifically examines the agreement or disagreement of fertility intentions among couples. To this end, we have restricted our sample to include individuals within the age range of 18–44 years who were part of a couple at the time of the interview. These criteria align with standard practices in fertility research, as agreement on childbearing inherently requires both partners to be in a relationship and of reproductive age. We also excluded respondents if they or their partners were infertile, already pregnant during the interview, or if data were missing on their fertility plans or birth country (of either member of the couple). We ended up with 3133 couples, of whom about 69% had both partners as natives, 15% were migrants, and 16% were in mixed unions (see Table A1 in appendix).

### Dependent Variables

Given the dyadic nature of reproduction, couples are the most suitable context for investigating fertility decision-making; however, very few representative surveys collect information on both parents separately (Testa, 2012). We handle this limitation by deriving our dependent variable from several targeted survey questions regarding the fertility intentions of the partners, including the perception of the partner’s answer (Testa, 2012). Respondents were asked if they planned to have a(nother) child within the next 3 years, if they intended to have children eventually if not within that timeframe, and whether their partner desired to have a(nother) child. We synthesized responses from the first two questions to estimate the respondent’s lifetime fertility intentions and then juxtaposed this with their perception of their partner’s intention. This generated a categorical variable with three possible outcomes: mutual agreement to have a(nother) child (“AgreeYes”), mutual agreement to stop childbearing (“AgreeNo”), and disagreement on having a(nother) child (“Disagree”).

Furthermore, affirmative answers to the inquiry about actively trying to conceive a(nother) child with the partner were also categorized as “AgreeYes,” aligning with Neyer et al. (2022), which posits that current efforts to conceive imply intentions.

### Independent Variables

The main independent variable is couple’s migrant background and is based on the combination of country of birth of the partners. For the ego, we would be able to distinguish the macro area of origin, but unfortunately, for the partner we can only distinguish if she/he is born in Sweden or abroad. We also created a variable indicating if in the mixed couple the ego was native or migrant (mainly for a robustness analysis—Figs. 2 and A3 in appendix). Our other independent variables encompass demographic and socio-economic factors at the interview including the age class, education, employment status, parity, religiosity and attitude towards gender equality.^1^ Additionally, we control for couples’ characteristics like type of partnership (marriage vs. cohabitation) and age and educational differences between partners. Furthermore, the survey provides a rich set of indicators, which are used in the descriptive analysis to examine the composition of couples across migrant backgrounds (Table A2 in appendix). However, these indicators are not included in the multivariate analysis in order to avoid collinearity and achieve a parsimonious model.

### Methods

To address our research question, we use multinomial logistic regression to differentiate patterns of fertility intention agreement/disagreement among mixed and homogamous couples (both native-born and both migrants). We first run a model without controls (null model), and then with the full set of control variables. Both models are stratified by gender of the person reporting on the couple’s agreement/disagreement. We present the results as predicted probabilities, adjusting for whether either person in the couple has migrant background. We hold other covariates constant at their mean values to aid interpretability, following the approach recommended by Williams (2012). Following Goldstein and Healy (1995), we computed confidence intervals with a level of 83.5%. This approach ensures an average 5% level for Type I errors in pairwise comparisons of a collection of means. A similar approach has been used with survey data with a small sample size or when examining migrants (Alderotti et al., 2022, 2023).

## Results

### Descriptive Findings

The descriptive statistics presented in Table 1 show levels of agreement on fertility intentions (“AgreeYes,” “AgreeNo,” or “Disagree”) by migrant background of the couple and gender of the respondent. Across all groups and genders, the majority of couples are aligned on their fertility intentions. However, agreement levels vary significantly depending on whether the couple consists of two natives, two migrants, or is a mixed pair. Both-migrant couples show the highest agreement to have a(nother) child, regardless of whether the respondent is a man or a woman. When the respondent is a man, in a both-migrant couple, 36.9 per cent report agreement to have a(nother) child, compared to 25.9 per cent of both-native couples and 23.8 per cent of mixed couples. A similar pattern emerges among female respondents, with 38.1 per cent of both-migrant couples agreeing to have a(nother) child, compared to 26.3 per cent of both-native couples and 24.6 per cent of mixed couples. This pattern suggests that migrant couples, regardless of whether the man or the woman is responding, may have stronger alignment in their fertility intentions, possibly due to shared fertility expectations or cultural norms. In contrast, mixed couples report the lowest levels of agreement, with 23.8 per cent among men and 24.6 per cent among women, indicating greater variation in fertility expectations and family planning norms when partners come from different cultural backgrounds. Consistent with these findings, mixed couples also display the highest levels of disagreement, with 22.9 per cent among men and 25.9 per cent among women, compared to 17.4 per cent and 20.2 per cent among both-native couples and 13.2 per cent and 16.6 per cent among both-migrant couples. This pattern suggests that mixed couples may face greater challenges in reaching consensus on fertility intentions, likely due to differences in cultural backgrounds and family planning norms. Table 1 further illustrates how demographic, socioeconomic, and attitudinal characteristics vary by migrant background and gender. Migrant couples tend to be younger, more likely to be married, have larger age gaps between partners, and hold more traditional gender role attitudes. In contrast, native–native couples are generally more educated, more likely to cohabit rather than marry, and exhibit higher employment rates. Mixed couples tend to display intermediate characteristics but with greater variability, particularly in terms of age, education, and partnership status. Gender asymmetries are particularly evident in mixed couples, where men and women differ more in age, employment status, gender role attitudes, and religiosity compared to both-native and both-migrant couples (Table 1). Among mixed couples, men report higher levels of disagreement on fertility intentions than women, suggesting that differences in cultural background may play a role in shaping fertility decision-making. Additionally, comparing men and women, migrant couples show larger differences in age, and employment, while mixed couples have the most pronounced gender gaps in fertility agreement, gender role attitudes, and religiosity. Women in mixed unions tend to be more traditional and religious than their male partner (see Table 1).

**Table 1:** **Characteristics of the sample by couples' migrant background and gender.** Source: GGS our elaboration, weighted results

	Men				Women			
	Both natives	Mixed couples	Both migrants	Total	Both natives	Mixed couples	Both migrants	Total
Agreement								
AgreeYes	25.9	23.8	36.9	27.1	26.3	24.6	38.1	27.8
AgreeNo	56.7	53.3	49.8	55.2	53.5	49.6	45.3	51.6
Disagree	17.4	22.9	13.3	17.7	20.2	25.9	16.6	20.6
Age								
18–24	8.3	9.5	9.0	8.6	15.7	13.9	6.0	13.9
25–29	14.2	11.3	5.5	12.5	14.9	14.7	12.0	14.4
30–34	21.8	21.2	18.5	21.3	24.3	24.9	24.5	24.4
35–39	17.8	17.0	22.3	18.3	15.7	15.9	21.5	16.6
40–44	23.3	20.8	24.9	23.1	17.8	18.3	20.6	18.3
45–49	14.6	20.2	19.8	16.3	11.6	12.4	15.3	12.3
Education								
9 years or less	9.2	7.3	14.3	9.6	10.7	8.5	10.5	10.3
Upper secondary	45.0	28.2	15.9	38.3	36.0	25.5	24.1	32.5
Post-secondary	45.7	58.8	52.6	48.8	53.2	62.0	47.2	53.7
Missing	0.2	5.7	17.2	3.4	0.2	4.0	18.2	3.5
Partnership								
Married	39.8	44.5	64.3	44.0	37.2	40.4	66.9	42.2
Cohabiting	47.0	33.4	16.5	40.6	45.5	31.1	23.6	39.8
Non-cohabiting	13.1	22.1	18.2	15.3	17.3	28.3	8.5	17.8
Unclear cohab. status	0.1	0.0	1.0	0.2	0.0	0.2	1.1	0.2
Age difference								
Ego younger	34.6	31.2	28.5	33.2	75.7	82.3	79.0	77.3
Same	17.0	16.8	14.8	16.7	11.9	6.7	9.8	10.7
Ego older	48.1	51.1	53.5	49.3	11.5	10.7	10.1	11.2
Missing	0.3	0.9	3.2	0.8	0.8	0.4	1.1	0.8
Education differences								
Ego less	29.5	19.1	12.6	25.4	14.3	17.0	16.2	15.0
Same	63.6	61.6	57.7	62.4	63.8	61.1	48.2	61.0
Ego more	6.4	12.3	9.7	7.8	21.5	17.2	14.3	19.7
Missing	0.6	7.0	20.1	4.4	0.5	4.8	21.3	4.4
Empolyment								
Employed	85.1	81.5	80.2	83.8	71.5	66.4	54.0	68.0
Student	9.3	14.4	10.7	10.3	16.3	14.9	15.0	15.9
Other	5.7	4.1	8.2	5.8	12.2	18.6	31.0	16.1
Missing	0.0	0.0	0.9	0.1				
Parity								
Childless	35.4	42.0	37.9	36.8	38.0	42.6	30.3	37.6
Parent	64.6	58.0	62.1	63.2	62.0	57.4	69.7	62.4
Gender role: mother working								
Other	94.6	92.8	73.4	91.4	96.8	92.8	76.8	93.1
Agree or more	5.4	7.2	26.6	8.6	3.2	7.2	23.2	6.9
Religiosity								
Not at all	52.0	45.6	25.9	47.3	39.4	36.1	18.7	35.7
Other	48.0	54.5	74.2	52.7	60.6	63.9	81.3	64.3

Additionally, Table A2 in appendix presents household and relationship characteristics by migrant background and gender. Language spoken at home varies significantly, with nearly all both-native couples speaking Swedish (about 99%), whereas a large proportion of both-migrant couples primarily speak other languages (80.2% of men, 84.9% of women). Religious service attendance is highest among migrants, particularly among women, 46.6% of women in both-migrant couples report attending religious services frequently, compared to only 30.1% of women in native–native couples. Regarding birth region, both-migrant couples display diverse origins, with a substantial presence of individuals from MENA, Eastern Europe, Asia, and Latin America. Relationship satisfaction is generally high across all groups, particularly among men in migrant couples. However, patterns of disagreement about money, in-laws, and childrearing differ by migrant background and gender. Men and women in native–native couples report the highest levels of agreement, while disagreement is more frequent among migrant and mixed couples. Notably, women in migrant couples report significantly more disagreement than men in similar migrant couple setups, suggesting potential power imbalances in decision-making. Communication styles also vary, with both-migrant couples reporting the lowest levels of calm discussion during conflicts. These descriptive patterns suggest that agreement/disagreements should be examined both by genders of the person reporting the intentions for the couple, and by native, migrant and mixed couples separately.

### Multinomial Regression

**Fig. A1:**
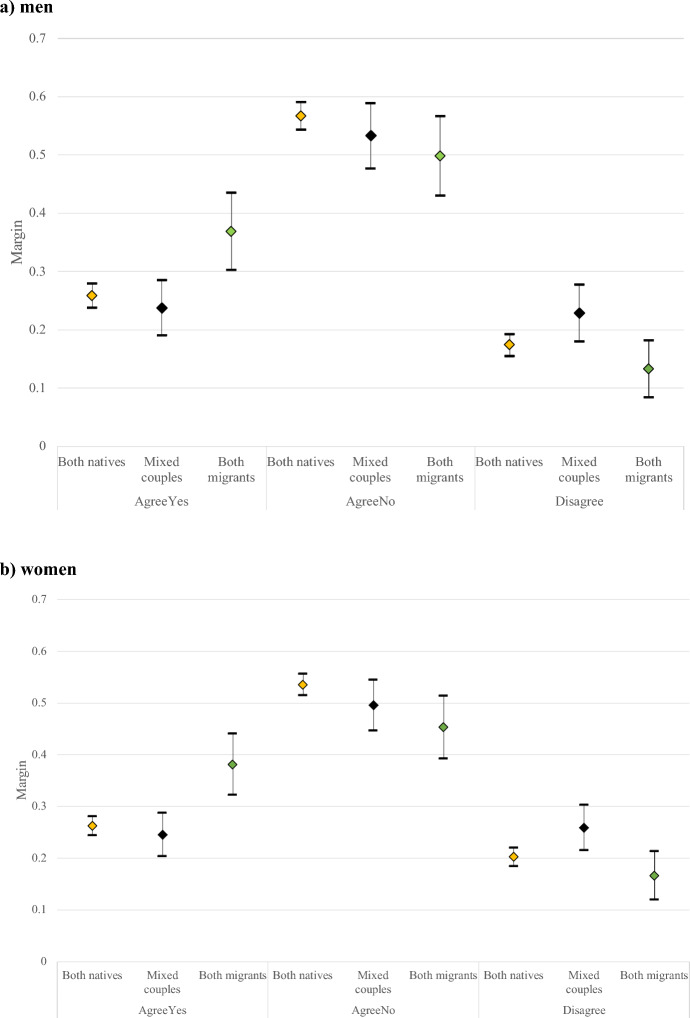
Predicted Probabilities of Fertility Intentions by Couples’ Migrant Background in Sweden by gender—Null Model. Source: GGS our elaboration, weighted results. *Note* mutual agreement to have a(nother) child (“AgreeYes”), mutual agreement to stop childbearing (“AgreeNo”), and disagreement on having a(nother) child (“Disagree”)

When we ran our multinomial logistic regression without any controls—Null Model (Appendix Figure A1)—we found very similar results across gender of the respondent to what we saw in the descriptive analysis. The null model shows that most couples agree to stop childbearing (although this difference is not significant for women in a migrant couple who agree to continue), in line with the current trend of decreasing fertility in Sweden. Among couples, despite slightly overlapping confidence intervals, mixed couples are positioned between those where both partners are either natives or migrants. Among couples that decide to have another child, migrants express the highest agreement, compared to native or mixed couples.

Differences between mixed and native couples are not significant. Among couples who disagree, mixed couples stand out, while migrant couples disagree less. However, when we control for the different composition of the couples, some gender differences emerge.

**Fig. 1:**
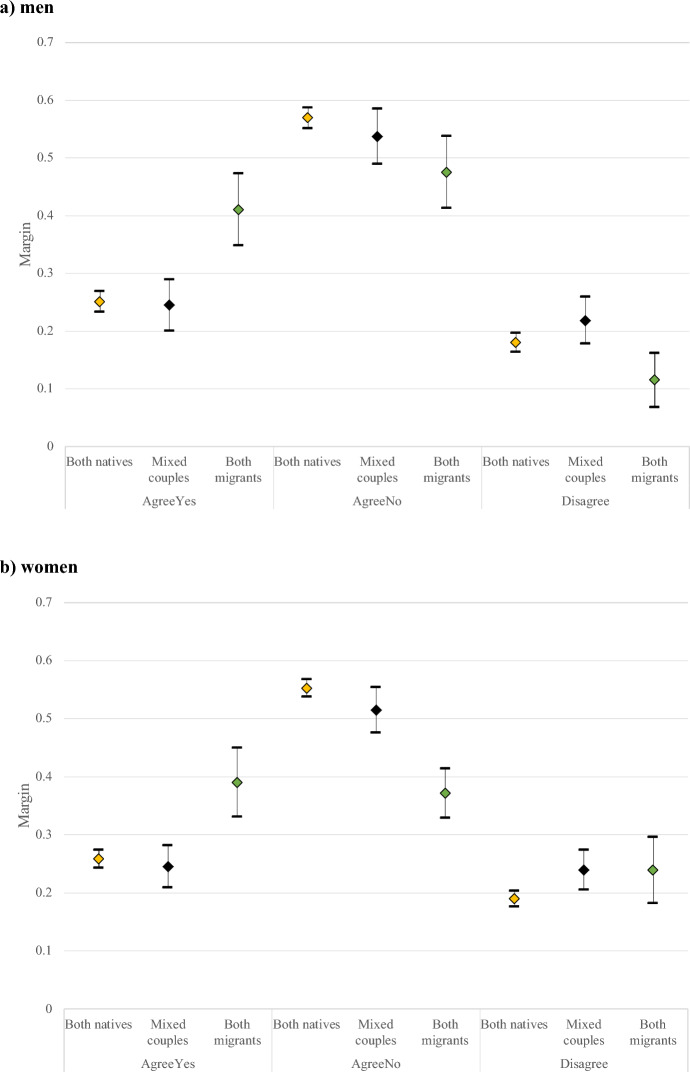
Predicted Probabilities of Fertility Intentions by Couples’ Migrant Background in Sweden—Full model. Source: GGS our elaboration, weighted results. Note: mutual agreement to have a(nother) child (“AgreeYes”), mutual agreement to stop childbearing (“AgreeNo”), and disagreement on having a(nother) child (“Disagree”). We controlled for age class, education, employment status, parity, gender of the respondent, for type of partnership, age and educational difference between partners, religiosity and gender equality

In the full model, we introduce all the controls in one step (Fig. 1). Our results on the predicted probabilities of agreeing or disagreeing to have a(another) child are quite stable. Among mixed couples both men and women, the highest probability is to agree to stop, with no statistical differences between agreeing to have a(another) child or disagreeing on future fertility plans. Among native couples as well, despite the gender of the ego, the highest probability is to agree on stopping: however, agreeing to have a(another) child is still slightly higher than the disagreement. Meanwhile, among migrant couples, it is confirmed that the probability of disagreement is lower, but there are no significant differences in the probability of agreeing to stop or having a(another) child, especially when women are reporting the intentions.

Results regarding agreement patterns show a slight difference by gender of the respondent. The agreement of having a(another) child is highest among couples where both individuals are migrants, with no difference between native or mixed couples or men and women respondents. Conversely, agreement to not have a(nother) child (AgreeNo), is highest among native Swedish and mixed couples, but significantly lower among individuals in a migrant couple only if the female is reporting the couple’s intentions. Disagreeing on whether to have another child (Disagree) is somewhat lower among migrant couples when men are reporting on the intentions) compared to native and mixed couples. Disagreement is also significantly lower for women in native couples compared to those in mixed couples.

In Table A3, in appendix, we present also the relative risk ratios (RRR) from the full model. Even if our main interest was analysing the differences in agreement/disagreement of fertility intention by couples’ migrant background, we would like to highlight the importance of individual socio-economic characteristics in comparing in particular the most common outcome “agree to have a(another) child” with agree to stop and disagreement. As expected, age plays an important role, particularly in the agreement to stop fertility. Highly educated individuals have a higher RRR of agreeing to have another child. Both age and education differences between partners’ matter. When the ego is a woman, higher education increases the RRR of agreeing to stop, whereas being younger decreases the RRR. Among men, having equal or higher education increases the RRR of disagreement, while being older decreases the RRR of agreeing to stop. Childless individuals tend to agree more on having another child, while parents agree more on intending to stop. Non-cohabiting parents have a higher RRR of disagreement. Gender role attitudes are more important for men, with more traditional gender role attitudes reducing the RRR of agreement on stopping. Religiosity is more influential for women, where higher religiosity lowers the RRR of agreeing to stop.

#### What About the Ego?

**Fig. 2:**
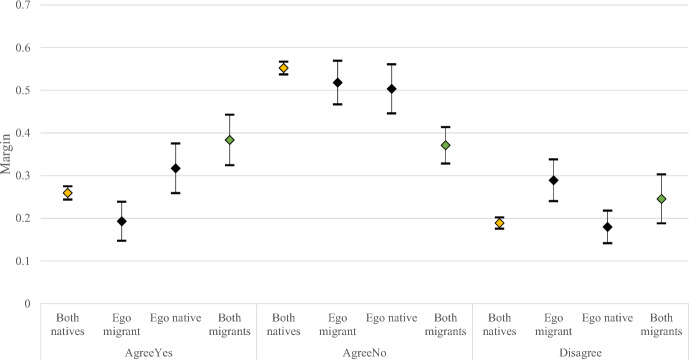
Predicted Probabilities of Fertility Intentions by Couples’ Migrant Background in four categories—Women, Full model. Source: GGS our elaboration, weighted results. Note: mutual agreement to have a(nother) child (“AgreeYes”), mutual agreement to stop childbearing (“AgreeNo”), and disagreement on having a(nother) child (“Disagree”). We controlled for age class, education, employment status, parity, gender of the respondent, for type of partnership, age and educational difference between partners, religiosity and gender equality

**Fig. A2:**
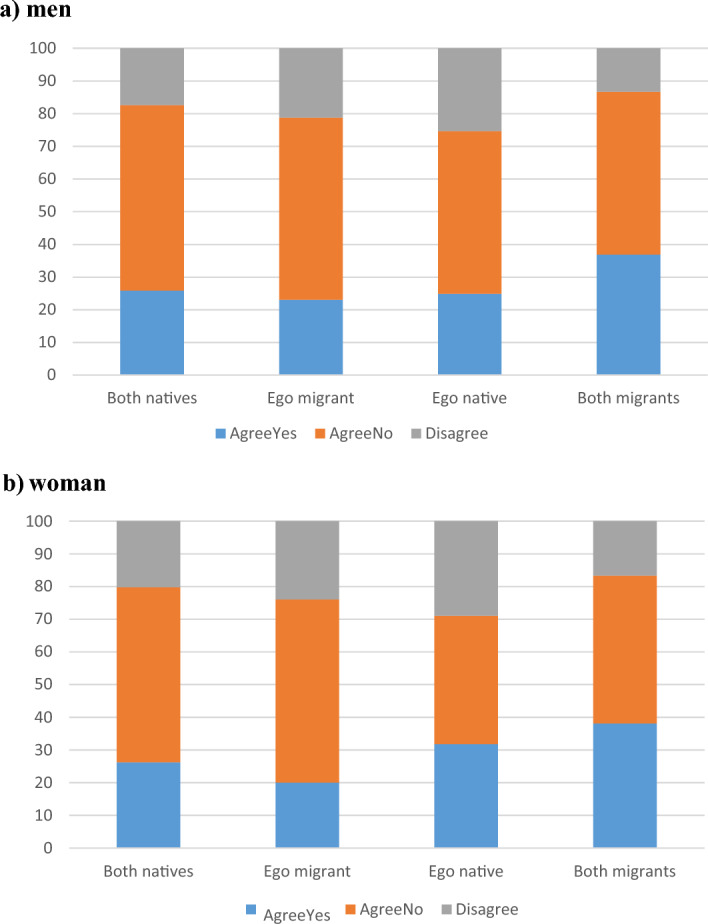
Percentage of agreement/disagreement by couples’ migrant background with four categories by gender. Source: GGS our elaboration, weighted results

**Fig. A3:**
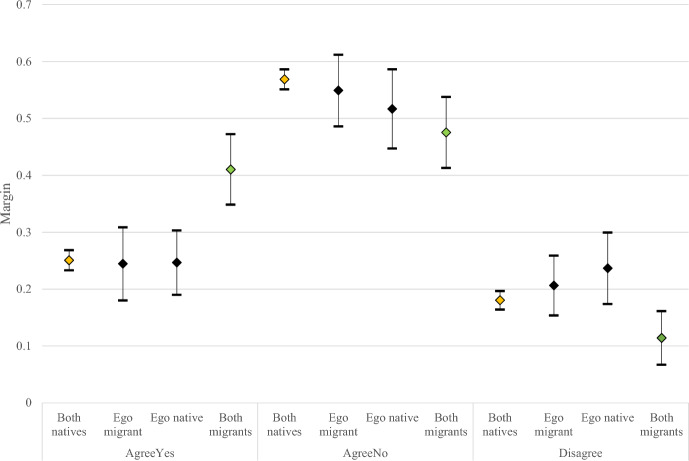
Predicted Probabilities of Fertility Intentions by Couples’ Migrant Background four categories: where respondent is a man. Source: GGS our elaboration, weighted results. *Note* mutual agreement to have a(nother) child (“AgreeYes”), mutual agreement to stop childbearing (“AgreeNo”), and disagreement on having a(nother) child (“Disagree”). We controlled for age class, education, employment status, parity, gender of the respondent, for type of partnership, age and educational difference between partners, religiosity, and gender equality

Some of the gender differences that persist after controlling for the independent covariates could be due to the different distribution of area of origin by gender across migrant backgrounds. Unfortunately, as mentioned before, lack of data means we cannot control for this aspect. However, as a robustness check, we split individuals in mixed couples by country of origin (Figure A2, appendix) (native vs. migrant of the ego). Among women (Fig. 2), but not men (Figure A3, appendix), couples in a mixed union where the ego is a native seem to agree more on having a(nother) child and disagree more compared to when the ego is a migrant, although this finding is limited by the small sample size. In our statistical models, we find that the migrant background of the ego matters only among women. A native ego is significantly more likely to report that the couple is agreeing to continue than a migrant ego. Additionally, a migrant ego is more likely to report disagreement than a native ego.

## Summary

Overall, migrant couples exhibit the strongest fertility agreement, whereas mixed couples show the highest disagreement and the most pronounced differences between men and women. The findings suggest that cultural background, gender norms, and selection effects may influence fertility decision-making and couple dynamics. Specifically, our results indicate that the higher disagreement observed in mixed couples likely reflects compositional/selection effects, as individuals who choose partners from different backgrounds may have distinct characteristics compared to those who partner with co-ethnics. Moreover, the stronger fertility agreement among migrant couples’ points to the role of cultural background, while the gender differences in reporting highlight the importance of gender norms in shaping reproductive decision-making. Descriptive results also underscore cultural and behavioural differences in family life and relationship dynamics across different couple types, particularly in terms of language, gender roles, disagreement patterns, and communication styles. Overall, our multivariate analysis suggests that when we control for socio-economic composition of the couples, some of the differences between couples in agreement are no longer significant. However, in the full model, some differences persist, but these are small in magnitude and varies by the respondent’s gender. Men in mixed couples disagree more than men in couples where both partners are migrants, but not significantly more than those in a Swedish couple. Women in mixed couples report significantly more disagreement only compared to those in a native Swedish couple. Moreover, we find that there are significant differences among mixed couples when the respondent is a migrant or a native woman. The differences between gender of the respondent might be related to the migrant composition (discussed further below in the limitation section). When exploring agreement for a positive fertility intention, migrants in Sweden are more likely than both native Swedish couples and mixed couples to agree to have another child. This echoes the actual fertility levels, which are higher among migrants than among ancestral Swedes (Mussino et al., 2021a, 2021b), and may reflect a consensus of both partners. Importantly, there are some differences between agreeing on a positive intention and agreeing on a negative intention across our couple groups. For agreeing to stop, mixed couples fall in between native Swedish couples and migrant couples, but results are significant only for women.

## Discussion and Conclusion

Recent research on partner choice in Europe suggests that assumptions about couple similarity may no longer be valid, given the increasing heterogeneity in couple composition and intermarriages (Blackwell & Lichter, 2004; Schwartz & Mare, 2005). With this background, we have examined agreement and disagreement on fertility intentions among couples in Sweden by migrant background. To our knowledge, this is the first study to deploy data on agreement and examine couple differences across migrant status. Sweden is a perfect case study with its significant and varied immigrant population. About a quarter of its residents are either migrants or having at least one migrant parent. The diversity in backgrounds is likely to influence family dynamics and fertility intentions in ways that differ from the native population. Although Sweden historically had relatively high fertility rates, there has recently been a decline to a record low.

Overall, our data show that: 1) It is informative to examine the direction (agreeing to continue or agreeing to stop) of short-term fertility intentions. Agreement to not have a(nother) child was highest among native Swedish couples, followed by mixed couples, and lowest among migrant couples. Interestingly, the full model with all controls did not alter the patterns much from the null model, suggesting that the observed differences in fertility agreement across couple types are robust and not merely artefacts of sociodemographic composition. 2) The migrant background of the couple matters in understanding agreement/disagreement on short-term fertility intentions. 3) The main results are generalizable for both men and women. However, for mixed couples, there are some gender differences in whether a male or female is the respondent. There are also probably some selection factors at play in who enters a mixed union. Specifically, native women who partner with migrant men appear more likely to report agreement on continuing childbearing than migrant women partnering with native men. This suggests that native women who select migrant partners may be more family-oriented or open to larger families than the average native woman, while migrant women who partner with native men may be more assimilated to Swedish fertility norms. These selection effects underscore how partner choice itself reflects underlying values and preferences that subsequently shape fertility decision-making.

While we note that the magnitude of the effects is rather slight, the results that mixed couples disagree more are in line with the vast literature on higher dissolution risks among intermarried/mixed couples (e.g. Dribe & Lundh, 2012). It is plausible that couples who hold different views on family size or timing of parenthood would face stress that could lead to relationship break-down. However, reverse causality is also possible if differences in values are associated with other stressors that mean that having a(nother) common child is less desirable.

One important limitation of this study is fact that the respondent report on their partner’s fertility intentions. Ideally, both partners should have been interviewed independently (Thomson, 1997; Thomson & Hoem, 1998), which could ease the interpretation of conflicting desires (Becker, 1981). However, due to data limitations, we are not the first to use perceived partner intentions. Unfortunately, with these GGS data, we cannot test how well perceived information on partner intentions predicts actual intentions. Fortunately, we can rely on previous conclusions. Testa (2012) suggests that the bias introduced by using partners’ perceived fertility desires as proxies for their actual fertility desires is not particularly high. In her paper, this means that real disagreement, not just perceived disagreement, is the reason for continuing to use contraception among conflicting couples. However, we hope that in the future more surveys will interview both partners separately.

Another limitation of our study includes a relatively small sample size. This is a common issue for surveys, especially those attempting to cover migrant populations. For instance, there is a difference in the number of couples in the GGS who are native, immigrant, and mixed. This means that in comparing native and migrant/mixed couples, caution should be exercised. A non-significant result for the mixed/migrant group may simply reflect fewer men and women in these groups.

We also have to keep in mind that the distribution of origin is quite diverse among couples where both partners are migrants and among mixed couples (as shown in Table A2 in appendix). Agreement among mixed couples could be higher if the migrant partner comes from a country with higher value similarity to Sweden. But unfortunately, we cannot test the role of socialization among migrants who come from another Nordic country versus a country in e.g. the Middle East. This is because for the partner, the data only distinguish between whether he/she is born in Sweden or abroad (not which country). For the ego, we only have access to country of birth and not ethnicity or language skills, factors that may all play a part in holding similar values in a relationship.

The findings reported here—higher levels of disagreement among mixed couples—are not necessarily generalizable to other contexts, including other European welfare states. Selection into a mixed couple may vary between different host countries; the degree of difference in socialization, integration possibilities, and social policies for family life may also differ. These factors may also influence whether a migrant chooses a particular host country (i.e., whether there is positive selection vis-à-vis childbearing norms for migrants who migrate to a specific country). While this study only covers one host country, it does cover a diverse range of mixed as well as migrant couples. Sweden has an exceptionally varied migrant population, and significant differences in childbearing behaviour exist between migrants from different continents (see Table A2). Therefore, Sweden is well positioned to shed light on whether couples with different socialisation during childhood indeed have greater dissimilarity in fertility intentions than those with more similar origins. However, the small number in our sample does not allow us to analyse differences by country of birth. This also hinders any examination of whether migrant women from contexts where female agency is lower overall would be less able to state their own intention in the case that it would divert from the intention of their male partner. Future studies should attempt to test whether mixed couples in other settings and mixed couples with different levels of value similarity (at the group level) differ in their agreement on continued childbearing. Nevertheless, this study provides a novel glimpse of couple dynamics and fertility agreement based on the Swedish setting and as such is something for other studies to build on once the data necessary is available.

We believe that agreement of fertility intentions is a pertinent topic, not just for wellbeing of individuals within couples, but also in the context of falling fertility levels. In Sweden as in many other European countries, fertility is now at record low levels and more research with a couple perspective ought to be conducted to understand the views and attitudes underlying this behaviour change. Policy makers concerned with declining fertility trends could benefit from understanding the couple-level dynamics of reproductive decision-making. Our findings suggest that examining whether childbearing intentions are aligned between partners provides valuable insights into fertility patterns. Specifically, knowing whether declining fertility stems from unanimous decisions to delay or forego parenthood, or from disagreement between partners, could inform more targeted and effective family policies. This couple-centred approach acknowledges that fertility outcomes result from negotiated decisions rather than individual preferences alone. Beyond fertility, agreement on fertility intentions is also crucial for other demographic outcomes as they might impact division of labour, childcare, parental leave uptake and ultimately relationship satisfaction and stability.

Overall, the aim of this study was not to explore whether agreement is associated with a higher likelihood to actually have a(nother) baby within the near future. Nor have we explored whether a negative intention is likely to be realised. Future studies should explore these questions, as there may be differences between couple’s abilities to realize intentions depending on migration background. Examining fertility intentions among couples with different compositions is important, both because mixed couples today make up a larger proportion of the entire population, and in the light of falling fertility across many countries in Europe and beyond.

## Data Availability

The data that support our findings are available from GGP. Restrictions apply to the availability of these data, which were used under license for the current study, and so are not publicly available.

## Appendix

See Tables A1, A2, A3 and Figs. A1, A2, A3.

**Table A1:** Distribution of the Dependent and Independent Variables: Percentages and Sample Sizes Source: GGS our elaboration, weighted results

	Freq.	Percent
Migrant Background		
Both natives	2165	69.12
Mixed couples	509	16.24
Both migrants	459	14.64
Agreement		
AgreeYes	860	27.45
AgreeNo	1671	53.33
Disagree	602	19.23
Age		
18–24	357	11.38
25–29	423	13.5
30–34	718	22.93
35–39	545	17.4
40–44	645	20.6
45–49	445	14.19
Education		
9 years or less	312	9.96
Upper secondary	1103	35.21
Post-secondary	1609	51.35
Missing	109	3.48
Partnership		
Married	1349	43.06
Cohabiting	1258	40.16
Non-cohabiting	520	16.59
Unclear cohab. status	6	0.19
Age difference		
Ego younger	1764	56.29
Same	425	13.56
Ego older	920	29.35
Missing	25	0.79
Education differences		
Ego less	626	19.98
Same	1,932	61.66
Ego more	439	14.01
Missing	136	4.36
Empolyment		
Employed	2,367	75.54
Student	414	13.22
Other	350	11.18
Missing	2	0.06
Parity		
Childless	1,166	37.21
Parent	1,967	62.79
Gender		
Male	1,493	47.65
Female	1,640	52.35
Total	3,133

**Table A2:** Household and Relationship Characteristics by Migrant Background and Gender. Source: GGS our elaboration, weighted results

Both natives		Men			Women			
		Mixed couples	Both migrants	Total	Both natives	Mixed couples	Both migrants	Total
Language spoken home								
Swedish	98.9	69.4	19.8	83.1	98.8	74.8	15.1	82.0
Other	1.1	30.1	80.2	16.8	0.9	25.2	84.9	17.7
Missing	0.1	0.5	0.0	0.1	0.3	0.0	0.0	0.2
Relationship Satisfaction								
Other	10.0	8.3	6.3	9.2	10.1	13.4	10.1	10.7
Completely	90.0	91.7	93.7	90.8	89.9	86.6	89.9	89.4
Disagreement: money								
Other	94.0	96.8	98.4	95.1	93.8	89.8	91.9	92.9
Frequently or more	6.0	3.2	1.6	4.9	6.2	10.2	8.2	7.1
Disagreement: Relationship with inlaw								
Other	94.8	95.3	94.7	94.9	96.1	93.8	85.2	94.0
Frequently or more	5.2	4.7	5.3	5.1	3.94	6.25	14.84	5.99
Disagreement: Child raising issue								
Other	74.1	67.0	68.3	72.2	68.0	64.1	64.2	66.8
Frequently or more	25.9	33.0	31.7	27.8	32.0	35.9	35.8	33.2
Disagreement/resultion: discuss calmly								
Other	29.8	29.5	31.7	30.0	33.3	32.3	36.7	33.7
Frequently or more	70.2	70.6	68.3	70.0	66.7	67.7	63.4	66.3
Religious Service attendence								
Never	69.9	63.6	53.7	66.6	64.0	60.9	53.4	61.9
Other	30.1	36.4	46.3	33.4	36.0	39.1	46.6	38.2
Area of birth								
Sweden	100.0	40.9	0.0	76.6	100.0	38.8	0.0	74.7
Western Europe, North America	stra0l.i0a	27.2	26.1	8.0	0.0	21.7	14.3	5.8
Eastern European	0.0	6.5	20.8	4.0	0.0	17.1	34.6	8.1
MENA	0.0	8.1	20.6	4.2	0.0	3.3	19.5	3.5
Other Asia	0.0	5.8	18.6	3.5	0.0	15.0	14.8	4.7
Other Africa	0.0	2.1	10.7	1.8	0.0	1.7	11.7	2.1
Latin America & Carib	0.0	9.4	3.1	1.9	0.0	2.4	5.2	1.2

**Table A3:** Multinomial Regression on Fertility Intentions Among Couples in Sweden: Relative Risk Ratios (RRR). Source: GGS our elaboration, weighted results

AgreeYes	Men AgreeNo Disagree		Women AgreeNo Disagree	
	RRR Sign	RRR Sign	RRR Sign	RRR Sign
Age (ref 18–24)				
25–29	0.51	0.23 **	0.81	0.35 **
30–34	0.87	0.13 ***	1.76	0.20 ***
35–39	2.00	0.10 ***	6.66 ***	0.23 **
40–44	7 08 ***	0.27 *	24.02 ***	0.80
45–49	26.98 ***	0.31	68.56 ***	0.90
Migrant background (Both natives)				
Mixed couples	0.90	1.34	0.86	1.52 *
Both migrants	_0.37 ***_	0.29 *	_0.23 ***_	0.91
Education (ref 9 years or less)				
Upper secondary	0.62	0.58	1.02	0.77
Post-secondary	0.50	0.38 *	0.41	0.59
Education differences (ref ego less)				
Same	1.38	2.17 *	1.53	0.93
Ego older	0.83	2.61 *	2.41 *	1.06
Age difference (ego younger)				
Same	1.00	1.51	0.60 *	1.12
Ego older	0.52 ***	1.12	0.53 *	0.73
Empolyment (Employed)				
Student	1.64	1.90 *	1.45	1.82 *
Other	0.66	1.35	0.83	1.01
Parity (ref childless)				
Parent	_3.82 ***_	2.11 *	6.66 ***	1.19
Partnership (ref Married)				
Cohabiting	_0.47 ***_	0.86	0.56 **	1.01
Non-cohabiting	1.30	2.98 **	1.70	3.07 **
Gender role-mother working (ref Other)				
Agree or more	0.46 *	0.96	1.20	0.69
Religiosity (ref not at all)				
Other	0.76	0.88	0.74 *	0.88
Constant	1.52	1.82	0.27 *	1.74

^**^ < 0.001, ** < 0.01, *0.1

and Figs. .

## Footnotes

1 We use the variable “Do you agree or disagree with the following statement: A pre-school child is likely to suffer if his/her mother working? We categorized responses in *agree or more* vs *other (strongly disagree, disagree, no opinion).*
